# Feasibility and safety of setting up a donor breastmilk bank in a neonatal prem unit in a resource limited setting: An observational, longitudinal cohort study

**DOI:** 10.1186/1471-2458-11-356

**Published:** 2011-05-20

**Authors:** Irene Coutsoudis, Miriam Adhikari, Nadia Nair, Anna Coutsoudis

**Affiliations:** 1 Department Paediatrics and Child Health, Nelson R Mandela School of Medicine, University of KwaZulu-Natal, Durban, South Africa

## Abstract

**Background:**

The beneficial effects of human milk on decreasing rates of paediatric infections such as necrotizing enterocolitis (NEC) and sepsis have been clearly demonstrated. Donor breastmilk has been encouraged as the milk of choice when a mother's own breastmilk is not available. The objectives of this study were to assess feasibility of providing donor breastmilk to infants in a resource limited Neonatal Prem Unit (NPU). In addition we sought to determine whether donor breastmilk could be safely pasteurized and administered to infants without any adverse events.

**Methods:**

Low birth weight infants < 1800 g and under 32 weeks gestational age were followed up in the NPU over a 3 week period; feeding data and morbidity data was collected in order to determine if there were any adverse events associated with donor breastmilk. Samples of pasteurized breastmilk were cultured to check for any bacterial contamination.

**Results:**

191 infants met the inclusion criteria of whom 96 received their mother's own breastmilk. Of the 95 infants who were potentially eligible to receive donor milk, only 40 did in fact receive donor milk. There was no evidence of bacterial contamination in the samples analyzed, and no evidence of adverse events from feeding with donor breastmilk.

**Conclusion:**

It is feasible to supply donor breastmilk to infants in an NPU in a resource limited setting, however staff needs to be sensitized to the importance of donor breastmilk to improve uptake rates. Secondly we showed that it is possible to supply donor breastmilk according to established guidelines with no adverse events therefore making it possible to prevent NEC and other side effects often associated with formula feeding of premature infants.

## Background

The particular benefits of human breastmilk for preterm and term infants have been well described in medical literature [1]. Human milk provides important nutritional components, digestive enzymes, immunological factors, growth factors, and hormones that make it a clinical standard of care for preterm (including very low-birth-weight) and term infants [2]. The beneficial effects of human milk (fresh and pasteurized) on rates of pediatric infection such as necrotizing enterocolitis (NEC) and sepsis have also been clearly demonstrated [3-5]. Donor breastmilk has been encouraged as the milk of choice when a mother's own breastmilk is not available due to illness/infections, medications, or other social reasons [6]. Using human milk is of particular importance for preterm infants of HIV infected mothers as early introduction of formula feeds could be the source of allergens or contaminants. These allergens/contaminants in such an immature infant could provoke gut epithelial damage and therefore put the child at increased risk of breastfeeding transmission [7-9] once breastfeeding commences. Research from the United States indicates that even if donor milk is only half as effective as mothers' own milk in reducing infection and NEC in newborn infants, providing donated pasteurized breastmilk in a neonatal clinical setting is still more cost-effective than relying on preterm formula [10].

Because of the well documented benefits of donor breastmilk, a donor breastmilk bank was set up in the Neonatal Prem unit (NPU) of King Edward VIII Hospital (KEH) in Durban, South Africa at the beginning of 2009. KEH is a public hospital that serves a disadvantaged community and its NPU has an average intake of 180 infants per month. As part of the routine protocol in the NPU all mothers of HIV unexposed infants are encouraged to provide breastmilk to their infants. HIV infected mothers are routinely counseled on appropriate infant feeding for their situation according to WHO guidelines. HIV infected women who opt to breastfeed are taught how to heat treat their own expressed breastmilk (HTEBM) before they feed it to their infants. The new breastmilk bank that was opened a few months prior to the commencement of this evaluation study operates under the guidelines of the Human Milk Banking Association of South Africa (HMBASA) [11]. As it is unlikely that there will ever be an unlimited supply of donor milk, each child is assessed for their eligibility to receive donor milk. Preterm/low birth weight infants whose mothers are not able to supply their own milk are eligible to receive donor breastmilk if they are HIV infected and/or if they are at risk of NEC. The duration of donor breastmilk given to an infant depends firstly on the availability of donor breastmilk supplies and secondly, on the child's condition as assessed by the NPU doctor in charge. Donor mothers are sourced from the NPU and obstetric wards of KEH and they are healthy, HIV negative women (determined on 2 separate HIV tests) who are screened for absence of any lifestyle risks. All donor mothers in the unit use hand expression to express breastmilk into a 250 ml sterile glass jar which is then pasteurized by a breastmilk bank assistant, rapidly cooled and then decanted into a smaller sterile glass jar for storing. Following HMBASA guidelines, post-pasteurisation, an aliquot of milk is removed from the first donation of each new donor and is tested for microbial contamination. Each donor is given a donor number which is recorded on the bottle of milk together with the date of expression. Donor breastmilk is kept frozen in a -20°C chest freezer for up to 3 months after expression. Because the concept of donation of breastmilk was new in the hospital, when there were periods of insufficient supplies the breastmilk bank also sourced pasteurized donor breastmilk from a nearby NGO run community based breastmilk bank. This NGO run milk bank uses the same donor screening and bacterial screening practices, the only difference is that the milk is pasteurized by the Holder Pasteurisation Method using an automated commercial pasteurizer (Sterifeed S90).

Implementing a milk bank in this resource-poor setting presented multiple challenges particularly because of inadequate staffing and lack of funds for maintenance of equipment. Therefore a decision was made to use a method of pasteurization that would be more appropriate within these limitations. This pasteurization method, known as flash-heat, is a simple method which involves heating individual jars of breastmilk in a water bath. Flash-heat mimics commercial high-temperature short-time (HTST) pasteurization. This 'low-tech' method of pasteurization, has been documented to inactivate cell-free [12] and cell-associated HIV [13]; destroy pathogenic and non-pathogenic bacteria [14] and preserves the vast majority of vitamins [15], immunoreactive proteins and immunoglobulins [16].

There is skepticism among health care workers and policy makers in South Africa around feasibility and safety of human milk banks in this area mainly because of the fear of HIV transmission and the cultural acceptability. These concerns have severely hampered the use of donor breastmilk and there is therefore an urgent need for this information from South Africa to clarify these issues. This study hopes to provide some of this information which is in keeping with a resolution passed during the 61^st ^World Health Assembly Meeting in Geneva (May 2008) calling on the World Health Organization to support countries to conduct research on the feasibility of implementing breastmilk banks. There have been no prospective studies in South Africa examining the feasibility or safety of donor breastmilk supplied by breastmilk banks in NPUs. However there has been a report of anecdotal data suggesting that it was feasible and safe to provide donor breastmilk to infants during an outbreak of rotavirus [17].

The objectives of this study were twofold. First, we sought to address feasibility by documenting whether the medical staff would prescribe donor milk for infants; and whether eligible mothers in the unit would be prepared to donate breastmilk to the bank. We also investigated the feasibility of adhering to the HMBASA quality assurance guidelines for microbial testing of breastmilk. Secondly we sought to evaluate an aspect of the safety of providing donor milk by documenting clinical adverse events of infants fed on donor milk compared to formula milk. We did not seek to investigate whether infants fed on donor milk had a better outcome than those fed on formula milk as this has already been established as concluded in a Cochrane Review [5].

## Methods

### Study site, population and design

The study was an observational longitudinal cohort study. Mothers with low birth weight infants were recruited to participate in this study. The study population consisted of Low birth weight infants < 1800 g and under 32 weeks gestational age who were admitted to the NPU at KEH during the 6 month period June 2009 to December 2009. We selected 1800 g as an upper weight limit since larger babies were more likely to have abbreviated hospital stays and thus insufficient days for observation. We also particularly focused on infants < 1800 g and < than 32 weeks gestational age as they are at particular risk of developing NEC and other problems associated with formula feeds. Exclusion criteria included infants whose hospital stay was less than 3 days; infants with congenital abnormalities and infants who were only admitted to the unit > 24 hours after birth. Infants whose mothers were not able or willing to sign written informed consent were also not eligible for the study.

### Data collection methods and tools

Enrolment data was collected from the peripartum history and maternal medical history recorded in the hospital charts. During the infant's hospital stay morbidity and feeding data was collected. Frequency of neonatal complications during a 3 week observation period were recorded after clinical examination by the study doctor (IC) and from perusal of the clinical records. Infection was documented by the presence of clinical and laboratory signs of a systemic inflammatory response and/or positive cultures for pathogenic organisms at one or more of the following sites: blood, spinal fluid, urine, stool, respiratory secretions, umbilicus, or surgical wound. The morbidity was analyzed according to receipt of any donor milk vs receipt of formula milk only.

### Methodology for microbiology

An aliquot of 5 mls was removed from the first sample of pasteurized expressed breastmilk from each donor and was kept frozen at -20°C until it was analyzed.

Frozen samples were transported in batches to the National Health Laboratory Services at Inkosi Albert Luthuli Hospital, Durban. The methodology involved plating a 100 μl of the pasteurized milk sample onto MacConkey agar which was incubated for 24 hrs at 37°C. The following day a semi-quantitative count was performed. If any suspicious colonies were observed they were identified further.

### Statistical considerations

The study was analyzed using SPSS 15.0 (^© ^SPSS Inc.). Continuous variables were checked for normality of distribution and means/standard deviations (SD) are reported for the normally distributed variables and medians/inter-quartile ranges for the skewed variables. Counts and observed percentages are reported for the categorical data. Chi squared tests were used to compare the characteristics and complications present in the infants who received donor breastmilk and/or formula feeds.

### Ethical review

The study protocol was approved by the Biomedical Ethics Committee, University of KwaZulu Natal (ref: BE 176/08) and the King Edward V111^th ^Hospital Authorities.

Written informed consent was obtained from mothers for their infants' clinical records to be used in the study.

## Results

During the study period June to November 2009, a total of 191 infants met the inclusion criteria and were enrolled into the study. The baseline characteristics of the 191 mother/infant pairs who were enrolled in the study are shown in table 1. The mean duration of hospital stay ranged from 3 to 72 days and 83 infants (43.5%) actually had a hospital day of less than 3 weeks which was the expected period of study observation. Of the 191 infants, 96 received their mother's own breastmilk so potentially 95 infants were eligible to receive donor milk as a first feed. Only 16 received donor milk and the remaining 79 infants received formula milk as the first feed. However of the 79 infants who were started on formula milk a further 24 infants were changed to donor milk. Therefore of 95 infants who were potentially eligible to receive donor milk only 40 did in fact receive donor milk. In only one case was the reason due to mother refusing donor milk, in all the other cases it had not been offered as an option to the mother. The reasons for prescribing donor milk for these 40 infants is shown in table 2.

**Table 1:** Characteristics of the mothers and infants (n = 191)

Parameter		
**Maternal age (years): Mean (SD)**		26.51 (6.07)

**Maternal positive HIV status: number (%)**		100 (52.4)

**Twin delivery: number (%)**		43 (22.5)

**Current or treated TB: number (%)**		10 (5.24)

**Current or treated Syphilis: number (%)**		6 (3.1)

**Peripartum complications: number (%)**		169 (88.48)

**Caesarean delivery: number (%) **		99 (51.8)

**Gestational age (weeks): Median (IQR) **		32 (30-33)

**Small for gestational age infant: number (%)**		61 (31.9)

**Infant birth weight (kg): Mean (SD)**		1.38 (0.26)

**Duration of hospitalisation Median (IQR)**		22 (13-30)

**First feed used:**	**Formula: number (%)**	79 (41.4)
	
	**Breastfeed: number (%)**	81 (42.4)
	
	**HTEBM: number (%)**	15 (7.9)
	
	**Donor breastmilk: number (%)**	16 (8.4)

**Table 2:** Reasons for use of any donor breastmilk (n = 40)

Reason	Frequency
Insufficient breastmilk*	27 (67.5)

**Twins**	1(2.5)

**Not tolerating formula**	3 (7.5)

**Sick infant**	1(2.5)

**Mum sick/unable to breastfeed**	4 (10.0)

**Re-feeding after NEC related to formula**	1(2.5)

**Post c/s**	3 (7.5)

* According to mother and/or nursing staff

In terms of adverse events/clinical outcome, as can be seen in table 3, there were no differences in prevalence of neonatal complications in those infants who received any donor breastmilk vs. those who received only formula milk. Because of limitations in terms of a wide variation in the risk profile; and duration and type of feeds it is difficult to do these types of comparisons and we therefore also decided to capture details of individual case histories of some of the infants who received donor breastmilk in order to better capture the safety of donor breastmilk. Two of these are presented in summary form:

**Table 3:** Prevalence of neonatal complications and characteristics amongst the infants who received donor breastmilk and/or formula milk for > 24 hours (n = 106)

Complications (% within each type of feed)	Type of feeds received		
	
	Formula (n = 66)	Donor breastmilk (n = 18)	Both formula and donor (n = 22)
**Neonatal sepsis**	61(92.4)	18(100)	20 (90.9)

**Neonatal jaundice**	54 (81.8)	15 (83.3)	20 (90.9)

**NEC**	10 (15.2)	2 (11.1)	5(22.7)

**Abdominal distension**	22 (33.3)	7 (38.9)	7 (31.8)

**Feeds intolerance**	15 (22.7)	2 (11.1)	7 (31.8)

**Hypoglycemia**	1 (1.5)	0 (0)	1 (4.5)

**Pneumonia**	5 (7.6)	2 (11.1)	2 (9.1)

**Severe respiratory distress requiring CPAP or IPPV**	20 (30.3)	8 (44.4)	7 (31.8)

**Seizures**	3(4.5)	1(5.6)	1(4.5)

**Meningitis**	1(1.5)	0 (0)	2 (9.1)

**Nosocomial sepsis**	35 (53)	11(61.1)	20 (90.9)

**Severe anaemia requiring transfusion**	20 (30.3)	6 (33.3)	10 (45.4)

**Broncho-pulmonary dysplasia (BPD)**	8 (12.1)	4 (22.2)	4 (18.2)

**IVH**	6 (9.1)	0 (0)	6 (27.3)

**Superficial infections**	15 (22.7)	4 (22.2)	6 (27.3)

**Congenital syphilis**	1 (1.5)	0 (0)	1 (4.5)

**Duration of hospitalization (days): Median (Range)**	22 (5-71)	16.5 (6-44)	25 (11-72)

**Weight gain per week (kg): Median (Range)**	0.054 (-0.28-0.18)	0.059 (-0.06-0.23)	0.057 (-0.06-0.12)

*p values were not significant (< 0.05) for the differences between the three groups

Infant A was born at 32 weeks gestation (birth weight: 1.7 kg) to a 33 year old HIV negative, para 3 mother following a caesarian section for severe pre-eclampsia. Mother had left arm paralysis since adolescence following a head injury. She had chosen to breastfeed antenatally, however the nurses decided as she had a paralytic arm, she would be unable to hold her baby and started her baby on formula. NEC developed on day 2 following formula feeds (abdominal distension; feed intolerance; severe umbilical flare; dilated visible bowel loops; and pneumatoses on abdominal X-ray). Feeds were discontinued for 2 days, following which breastfeeding was initiated by the mother supported by the study doctor. Donor milk was given for four days while the mother's milk was coming down. NEC resolved after 6 days on treatment. Baby was doing well and discharged on breastfeeds and expressed breastmilk top-ups, 4 days after resolution of NEC.

Infant B was born at 29 weeks gestation (birth weight 1.1 kg) to a 41 yr old HIV negative, para 4 mother, with previous breast cancer and therefore not planning to breastfeed. Infant received formula milk feeds for the first 3 weeks of life and developed NEC (abdominal distension, vomiting, bloody stools and thickened bowel wall on abdominal X-Ray). He was taken off feeds for 5 days and then commenced on donor breastmilk for a further 3 weeks until discharge. Did extremely well on donor breastmilk and showed marked clinical improvement. In fact during the period of receiving donor breast milk the nursing staff attempted re-introducing formula feeds but these were not tolerated at all and were accompanied by projectile vomiting so were discontinued and donor breastmilk re-introduced.

We were successful in obtaining donors from amongst the mothers who were in hospital usually waiting for their infants to gain weight or get better. During the 6 month period of study we were able to recruit 35 donors. Because the concept of donation of breastmilk was new in the hospital when there were periods of insufficient supplies the breastmilk bank also sourced breastmilk from a nearby, NGO run, community based breastmilk bank. It was estimated that during the first 12 months of operation approximately 20% of the supplies were externally sourced whereas in the last 12 months (Jan 2010 to December 2010) the bank is operating completely on in-house donations of breastmilk.

Of the 35 in-house donors, 30 of the samples were analyzed for presence of bacterial contamination, post-pasteurization (flash heating). No bacterial contamination was found in any of the 30 samples analyzed.

## Discussion

This evaluation has shown that donor milk was in fact prescribed for 40 of the potentially 95 eligible infants. There were obvious missed opportunities which were due to medical staff not discussing with mothers that there was donor breastmilk available and that this was an option. We did not evaluate in each case why donor breastmilk was not prescribed when it was indicated but anecdotal evidence suggests that there were 2 main reasons. First, at the time of the study, where there was no routine nevirapine prophylaxis during breastfeeding, the standard NPU protocol was to prescribe preterm formula for HIV exposed infants whose mothers had either chosen to formula feed or who had chosen to breastfeed but were unable to provide the necessary milk. Second, because donor breastmilk was a very new option in the unit and because of the rotation of medical staff through the unit it takes a while to train all the staff and gain acceptance for donor breastmilk. Additionally it is easier and less time consuming for nurses and doctors to prescribe formula feeds compared to donor milk and the formula feeds are easier to access compared to the donor milk. In effect 42% of the eligible infants had donor breastmilk prescribed for them which we believe is an acceptable percentage for such a new concept. While this evaluation study was being conducted a separate acceptability study was also conducted in the unit. Results of this study showed that there were indeed concerns and lack of understanding around donor breastmilk which participants felt could be overcome with education and dissemination of information [18]. In response to the findings of the acceptability study the unit developed educational materials and resources and conducted educational sessions around donor breastmilk banking. Recent anecdotal data suggests that this education has resulted in the medical team being more convinced of the safety of donor breastmilk and more infants are now being offered donor breastmilk.

This evaluation has also shown that it is possible to supply donor breastmilk in neonatal prem units in resource poor settings with no adverse events experienced by the infants, therefore making it possible to prevent the NEC and other side effects often associated with formula feeding of premature infants. This also has important implications in terms of protecting and promoting breastfeeding and has the potential to play a role in decreasing infant mortality rate as has been shown in a country like Brazil which has reported dramatic decreases in its under-five mortality rate following the growth in donor breastmilk banking [19]. There are other obvious benefits of being able to supply donor breastmilk in settings with high HIV prevalence as the new WHO guidelines encourage breastfeeding by HIV infected women while the infants receive nevirapine prophylaxis [20]. There were obvious limitations to the study. Firstly the amount of time spent in hospital by each infant was variable so we tried to compensate for this by taking a set period of time which we felt would cover the majority of the infants viz. 3 weeks. However not all infants stayed in the unit for the full 3 weeks - 83 (43.5%) stayed for less than 3 weeks. An additional limitation of the study was that because we could not control the type of feeds each infant received; each infant may have received a variety of feeds, for example if mother is able to express a small amount of breastmilk which is insufficient for a full day's feed, the infant on that day may receive mother's own milk in addition to donor breastmilk. We were also unable to assess for differences between the infants who received formula only and those who received donor breastmilk only as the number of infants who received the donor breastmilk was small. Additionally we were not able to eliminate the strong possibility of reverse causality as neonatal complications often preceded and were the reason for the receipt of donor breastmilk. This was a significant limitation of the study and this could only have been assessed appropriately in a randomized controlled trial which would have been unethical.

Finally, although the small number of samples that were assayed showed no bacteriological contamination it is possible that with a much larger number of samples we would have detected some samples with bacterial contamination as was shown recently in an analysis from breastmilk banks in the US [21]. In particular breastmilk banks have shown concern that during the heating process there may be increased growth of *Bacillus *sp. [22]. However, while spore-forming *Bacillus *sp. may survive pasteurization, unlike cow's milk this is thought to be a rare contaminant of human breast milk [23]. Regardless, this type of contamination can be controlled by proper storage and handling after pasteurisation, which should prevent any *Bacillus *species that are present from growing. Some of these safeguards include, pasteurizing the milk as soon as possible after expression and then storing the sample frozen which will eliminate the majority of bacteria and thus avoid extensive growth of *Bacillus *during storage. Additionally pasteurized samples should remain covered and the lids should not be removed until feeding. It is also important to note that although *Bacillus *sp. may be a contaminant in food and dairy sources when it is present in breastmilk this danger is considerably attenuated because the medium of breastmilk unlike that of other food and dairy sources contains important factors that minimize bacterial growth. Furthermore it is important to balance the benefits of providing donor milk compared to the alternative of infant formula which has also been shown to be prone to contamination even with *Bacillus *sp [24].

We acknowledge that further investigation into cost-effective and simplified donor milk screening is warranted to allow for testing of each sample specifically for *Bacillus *sp., especially for low-cost settings desiring to utilize a simple method of pasteurization such as Flash-heat described here. However the current process of culture methods would be cost-prohibitive in resource poor settings. A methodology that may be more applicable in resource poor settings may be the system employed in the Brazilian banks in which a pH test is utilized to identify potential bacterial contamination [25]. We recommend that research is needed to develop a simplified low-tech tool to detect bacterial contamination such as a dipstick rapid diagnostic test. This would have important benefits for developing affordable quality assurance systems for breastmilk banks in resource limited settings.

## Conclusion

This study has shown firstly that it is feasible to access mothers who are prepared to dnate breastmilk and it is feasible for infants to receive donated breastmilk. Secondly we have shown that it is possible to supply donor breastmilk according to established guidelines in NPUs in resource poor settings with no adverse events, therefore making it possible to prevent NEC and other side effects often associated with formula feeding of premature infants. We believe this low-tech system of breastmilk banking can be reproduced in similar settings and would recommend that for successful scale-up, the endorsement and involvement of the Ministry of Health is essential as has been seen in the Brazilian system of breastmilk banking. Furthermore we recommend that low income countries should follow the Brazilian model of having a comprehensive, integrated breastfeeding promotion strategy including donor breastmilk banking with the ultimate aim of improving child survival. In South Africa especially, scale up of donor breastmilk banks would have a profound impact on breastfeeding promotion which is vital in order to improve infant survival rates. Furthermore this would assist to reverse current predictions that South Africa is unlikely to meet the millennium development goal 4 [26].

## Conflict of interests

The authors declare that they have no competing interests.

## Authors' contributions

IC conceived the study and all authors contributed towards planning and design of the study. IC together with a Zulu speaking assistant enrolled all the study participants and IC collected all the clinical data which was supervised by MA and NN. IC wrote the first draft of the paper and the other authors contributed to editing and finalizing the document. All authors read and approved the final manuscript.

## Pre-publication history

The pre-publication history for this paper can be accessed here:

http://www.biomedcentral.com/1471-2458/11/356/prepub

## References

[B1] Grulee CG Sanford HN Herron PH Breast and artificial feeding influence on morbidity and mortality of twenty thousand infants JAMA 1934 103 735 9

[B2] Lawrence RA Lawrence RM Breastfeeding: A guide for the medical profession 1999 5 St. Louise, Mosby

[B3] Lucas A Cole TJ Breastmilk and neonatal enterocolitis Lancet 1990 336 1519 23 10.1016/0140-6736(90)93304-8 1979363

[B4] El Mohandes AE Picard MB Simmens SJ Keiser JF Use of Human milk in the intensive care nursery decreases the incidence of nosocomial sepsis J Perinatol 1997 17 130 134 9134512

[B5] Quigley MA Henderson G Anthony MT McGuire W Formula milk versus donor breast milk for feeding preterm or low birth weight infants Cochrane Database Syst Rev 2007 4 CD002971 Oct 17 17943776 10.1002/14651858.CD002971.pub2

[B6] WHO/UNICEF Global strategy for infant and young child feeding 2003 Geneva, Switzerland: WHO

[B7] Coutsoudis A Pillay K Kuhn L Spooner E Tsai WY Coovadia HM Method of feeding and transmission of HIV-1 from mothers to children by 15 months of age: Prospective cohort study from Durban, South Africa AIDS 2001 15 379 87 10.1097/00002030-200102160-00011 11273218

[B8] Iliff PJ Piwoz EG Tavengwa NV Zunguza CD Marinda ET Nathoo KJ Moulton LH Ward BJ Humphrey JH ZVITAMBO study group Early exclusive breastfeeding reduces the risk of postnatal HIV-1 transmission and increases HIV-free survival AIDS 2005 19 699 708 10.1097/01.aids.0000166093.16446.c9 15821396

[B9] Coovadia HM Rollins NC Bland RM Little K Coutsoudis A Bennish ML Newell ML Mother-to-child transmission of HIV-1 infection during exclusive breastfeeding in the first 6 months of life: an intervention cohort study Lancet 2007 369 9567 1107 16 Mar 31 10.1016/S0140-6736(07)60283-9 17398310

[B10] Wight NE Donor Human Milk for preterm infants Journal of Perinatology 2001 21 249 54 10.1038/sj.jp.7200533 11533843

[B11] Guidelines for the Operation of a Donor Human Milk Bank in South Africa: Best practice for the collection, storage and handling of human milk 2008 Human Milk Banking Association of South Africa http://www.hmbasa.org.za

[B12] Israel-Ballard K Donovan R Chantry C Coutsoudis A Sheppard H Sibeko L Abrams B Flash-heat inactivation of HIV-1 in human milk: a potential method to reduce postnatal transmission in developing countries J Acquir Immune Defic Syndr 2007 45 318 323 17514015 10.1097/QAI.0b013e318074eeca

[B13] Volk ML Hanson CV Israel-Ballard K Chantry CJ Inactivation of cell-associated and cell-free HIV-1 by flash-heat treatment of breast milk J Acquir Immune Defic Syndr 2010 53 665 6 10.1097/QAI.0b013e3181ba47df 20335743

[B14] Israel-Ballard K Coutsoudis A Chantry CJ Sturm AW Karim F Sibeko L Abrams B Bacterial safety of flash-heated and unheated expressed breastmilk during storage J Trop Pediatr 2006 52 399 405 10.1093/tropej/fml043 17005732

[B15] Israel-Ballard K Abrams B Coutsoudis A Sibeko L Cheryk L Chantry C Vitamin Content of Breastmilk from HIV-1 Infected Mothers Before and After Flash-heat Treatment J Acquir Immune Defic Syndr 2008 48 444 9 10.1097/QAI.0b013e31817beb8d 18614920 PMC2896979

[B16] Chantry CJ Israel-Ballard K Moldoveanu Z Peerson J Coutsoudis A Sibeko L Abrams B Effect of flash-heat treatment on immunoglobulins in breast milk J Acquir Immune Defic Syndr 2009 51 264 267 10.1097/QAI.0b013e3181aa12f2 19421069 PMC2779733

[B17] Rinaldi M Brierley E Bekker A Donor breastmilk saved infant lives during an outbreak of rotavirus in South Africa Breastfeed Med 2009 4 2 133 134 10.1089/bfm.2009.0013 19500060

[B18] Coutsoudis I Petrites A Coutsoudis A Acceptability of donated breast milk in a resource limited South African setting Int Breastfeed J 2011 6 3 Feb 22 10.1186/1746-4358-6-3 21342496 PMC3049132

[B19] Almeida SG Dorea JG Quality control of banked milk in Brasilia, Brazil J Hum Lact 2006 22 335 9 10.1177/0890334406290099 16885494

[B20] WHO Antiretroviral drugs for treating pregnant women and preventing HIV infection in infants. Recommendations for a public health approach http://www.who.int/hiv/pub/mtct/antiretroviral2010/en/index.html PMC3535284 23293399

[B21] Landers S Updegrove K Bacteriological screening of donor human milk before and after Holder Pasteurisation Breastfeeding Medicine 2010 5 117 21 10.1089/bfm.2009.0032 20509779

[B22] Hanson ML Wendorff WL Houck KB Effect of heat treatment of milk on activation of *Bacillus *spores J Food Prot 2005 68 1484 6 16013392 10.4315/0362-028x-68.7.1484

[B23] Crielly EM Logan NA Anderton A Studies on the Bacillus flora of milk and milk products J Appl Bacteriol 1994 77 256 63 7989250 10.1111/j.1365-2672.1994.tb03072.x

[B24] Rahimifard N Fatholahzadeh B Pirali HM Nouri Z Saadati SH Zavar M Pirouz B Asghari SH Khesripour M Saberi S Bacillus cereus contamination in infant formula: a study in food and drug control laboratory Tehran University Medical Journal 2007 65 64 68

[B25] Novak FR Cordeiro DM The correlation between aerobic mesophilic microorganism counts and Dornic acidity in expressed human breastmilk J Pediatr (Rio J) 2007 83 87 91 10.2223/JPED.1589 17279292

[B26] Bryce J Daelmans B Dwivedi A Daelmans B Fauveau V Lawn JE Mason E Newby H Shankar A Starrs A Wardlaw T Countdown to 2015 for maternal, newborn, and child survival: the 2008 report on tracking coverage of interventions Lancet 2008 371 9620 1247 1258 18406859 10.1016/S0140-6736(08)60559-0

